# Supplementary material for: Investigation of Metabolic Resistance to Soybean Aphid (Aphis glycines Matsumura) Feeding in Soybean Cultivars
Source: Insects. 2022 Apr 5;13(4):356. doi: 10.3390/insects13040356 (PMC9025248; doi:10.3390/insects13040356)
Supplement: Supplementary file 1 [file insects-13-00356-s001.zip › insects-1621905-supplementary.pdf]

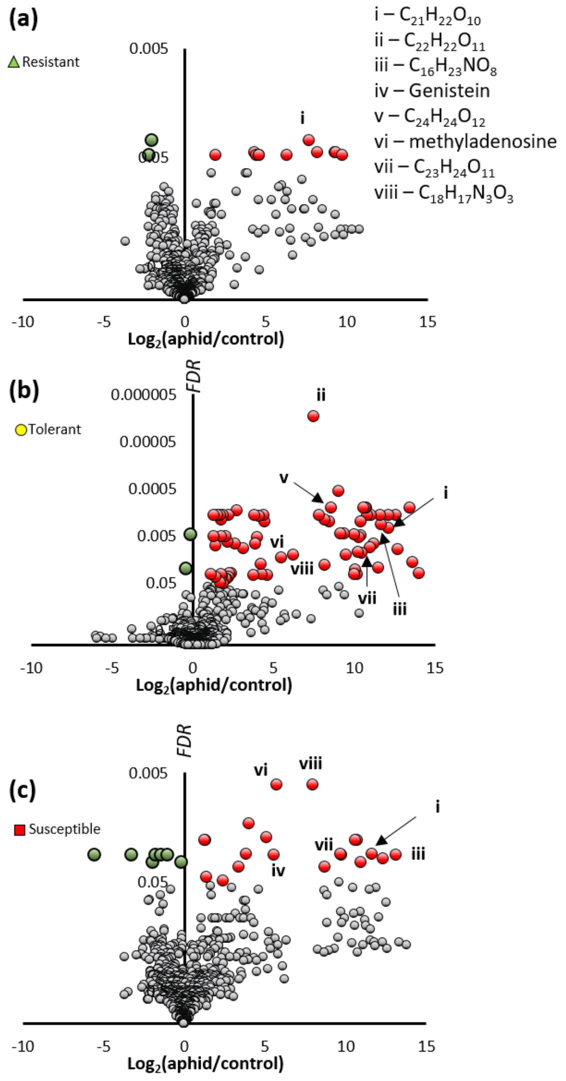

Figure S1. Volcano plot analysis of metabolite features of (**a**) R, (**b**) T, and (**c**) S cultivars altered by aphid infestation. Features in green were significantly (FDR <0.05) decreased upon aphid infestation and features in red were significantly increased by infestation.

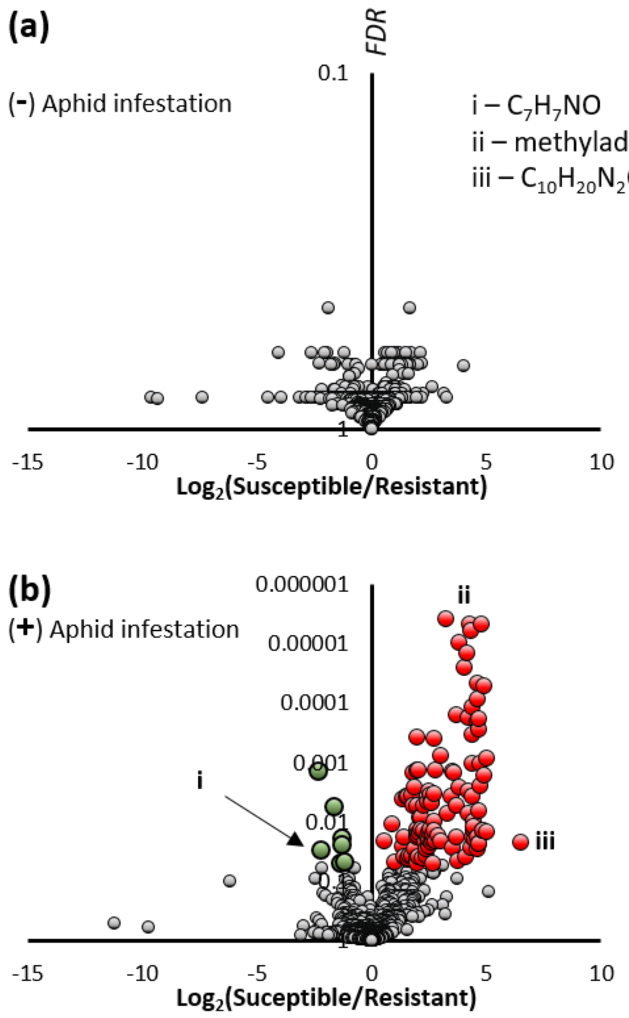

Figure S2. Volcano plot analysis of metabolite features of R and S cultivars in the (**a**) absence of aphid infestation and (**b**) presence of aphids. Features in green were significantly (FDR <0.05) higher in resistant cultivars and features in red were significantly increased in susceptible cultivars.

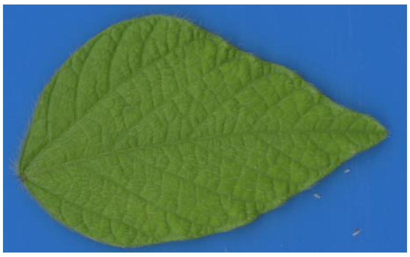

A

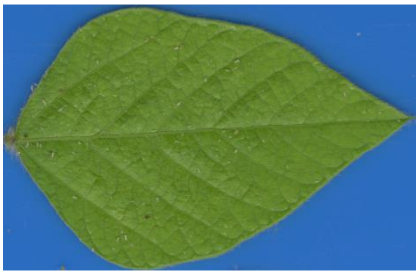

B

Figure S3. Control (**A**) and aphid-infested (**B**) AC Glengarry leaves from trifoliate 1.

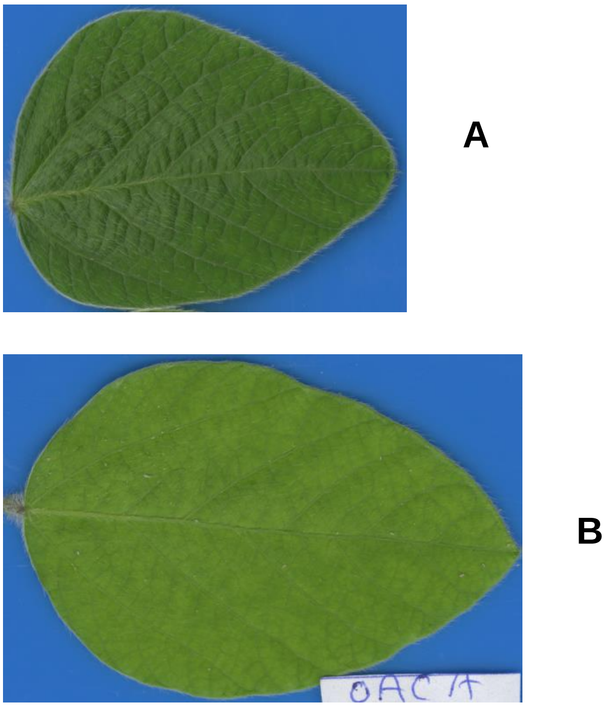

Figure S4. Control (**A**) and aphid-infested (**B**) OAC Avatar leaves from trifoliate 1.
